# Accurate Ab Initio Calculation of Molecular Constants

**DOI:** 10.6028/jres.103.013

**Published:** 1998-04-01

**Authors:** S. Kotochigova, I. Tupitsyn

**Affiliations:** National Institute of Standards and Technology, Gaithersburg, MD 20899-0001; Physics Department, St. Petersburg University, St. Petersburg, Russia, 198904

**Keywords:** AgH^+^ molecule, diatomic molecule, dissociation energy, equilibrium bond distance, molecular constants, OH molecule, valence-bond method, vibrational and rotational constants

## Abstract

Molecular constants have been computed for the ground states ^2^∏ of ^17^OH and ^1^Σ of ^107^AgH^+^. The valence-bond method and advanced computational technique were used to perform all-electron *ab initio* calculation of molecular electronic structures. The basic idea behind the model is to introduce the molecular wave functions in terms of Hartree-Fock many-electron atomic determinants. Full configuration interaction (CI) with nonorthogonal basis leads to the accurate calculation of molecular constants such as dissociation energy, equilibrium bond distance, vibrational and rotational constants with an agreement to the experimental data within a few percent.

## 1. Introduction

We present a new version of the valence-bond (VB) method, which is able to calculate the electronic structure of diatomic molecules. The first version of this *ab initio* method was developed by Heitler and London [1] and applied to the H_2_ molecule. The main idea of the method is to describe the molecular wave function as a linear combination of localized atomic wave functions. The new version of the method is oriented toward the calculation of many-electron molecules [2]. The self-consistent wave functions of the constituent atoms are constructed from determinants by using the Hartree-Fock (HF) method for non-relativistic atoms or the Dirac-Fock (DF) method for relativistic atoms. The construction of molecular wave functions from the many-electron atomic wave functions leads to the proper dissociation behavior. The total energy of the molecule as a function of inter-nuclear separation is presented as a power series in overlap integrals. This series converges quickly with atomic HF and DF functions.

The molecular basis set contains both occupied and unoccupied atomic HF or DF orbitals. Since the excited orbitals have a large radius and are not effective in describing the correlation and polarization in the molecule, we create additional basis functions made of localized orbitals, i.e., the Sturm’s wave functions [3]. This way of constructing the molecular wave functions avoids the need for large basis sets and ensures much shorter computation times as well as smaller errors in the calculation of the correlation expansion. The configuration interaction (CI) procedure, which takes into account the most important inner and outer shell correlation, simplifies and converges quickly as well, due to presence of HF or DF occupied orbitals.

The important advantage of this method is that the atomic core orbitals are not introduced by a pseudo-potential but calculated exactly. Although the inner-core orbitals do not affect significantly the molecular formation, the exact representation of the core orbitals is important for properties of molecules that depend on the inner core wave functions such as, for example, the hyperfine interactions. These core effects are especially pronounced for molecules with heavy atoms. In principle the Coulomb and exchange interactions can be calculated between all atomic orbitals, but this procedure can be limited to the most important orbitals to simplify the diagonalization procedure and save computer memory for the CI. The remaining orbitals will then form the core. A crucial step in the method is the calculation of many-center integrals using the sophisticated HF and DF functions. For the dimers the evaluation of the two-center integrals is simplified by using a reexpansion procedure [4]. In this procedure each of the two-center integrals is expanded in terms of one-center integrals located on the atomic centers. We have found that convergence of this expansion is accelerated by dividing the range of integration in two and considering only a reexpansion of the slowly varying part of the atomic wave function away from its nuclear center.

The formalism of the VB method is described in more detail in Ref. [2]. Here we discuss the computational details of a calculation of the molecular constants of the monohydrides OH and AgH^+^. The OH molecule is chosen because precise experimental data [5, 6, 7] as well as theoretical data [8] are available. To our knowledge no experimental data exist for the AgH^+^ molecule. However due to the fact that the hydrogen atom is slightly polarized by the Ag^+^ core, the bond is weak and an accurate determination of binding energy and bond length requires high precision of the theoretical method. Our AgH^+^ result is compared to the pseudo-potential calculation of Ref. [10].

## 2. Computational Details and Results

The first step of the calculation is related to computing the atomic wave functions. The HF functions are obtained with the one-electron center of gravity approximation and calculated for each atom of the molecule. Instead of taking large-radius unoccupied orbitals in building CI wave functions, we have added the Sturm’s orbitals. They are localized and create a nearly complete basis set. A multi-configuration CI technique is employed to calculate correlation corrections. For the OH molecule two CI basis sets were considered. The first has 38 configurations. The configurations are built from the 2*p*^4^ valence shell of oxygen, with additional single and double excitations of the 2*p* electrons into the 3*s*, 3*p*, 3*d*, 4*s*, 4*p*, and 4*d* orbitals, and the 1*s* orbital of hydrogen with excitation in the 2*s*, 2*p*, 3*s*, 3*p*, and 3*d* orbitals. The excited states in both atoms are described by Sturm’s wave functions. The 1*s*^2^2*s*^2^ shells of the O atom are treated as a core. The second basis has 262 configurations. The CI configurations include single-excitations of 1*s*^2^, 2*s*^2^, and 2*p*^4^ shells as well as double-excitation of the 2*p*^4^ shell where again these excitations are represented in terms of Sturm’s orbitals. Moreover both basis sets have configurations that represent the ionic states O^+^H^−^, and O^−^H^+^. The inclusion of excitation from the 1*s* and 2*s* orbitals of the oxygen atom in the second basis set leads to a better treatment of the core polarization. Table 1 shows that this substantially improves the agreement with experiment. The table includes the spectroscopical constants of the ground state of OH. Our best values disagree with experimental values by 0.2 % for the equilibrium internuclear distance *R*_e_, 2.7 % for the vibrational constant *ω*_e_, 0.4 % for the rotational constant *B*_e_, and 4 % for the dissociation energy *D*_e_. We want to point out that even with a very small basis set (38 configurations) we reached good agreement with experiment for the *R*_e_ and *B*_e_ parameters. Another theoretical result [8] obtained by a Hartree-Fock method using a *K*-functional treatment of correlation effects has a larger difference from the experimental values than ours. The origin of the disagreement in the dissociation energy of the VB and HF method seems to be related to the fundamental difference in constructing the molecular wave function. In the VB method the molecular wave function is constructed directly from many-electron atomic determinants with singly occupied atomic orbitals. For large internuclear separation this molecular wave function has the correct asymptotic behaviour and reduces to a product of the wave functions of the individual atoms. The HF method makes use of molecular orbital (MO) wave functions, which contain two electrons at all internuclear separations. In general, the one-determinant form of the HF method is a very poor approximation for large internuclear distances. As it is described in Ref. [9] the molecular wave function in Ref. [8] is introduced in a two-determinant form, which enables them to improve asymptotic behavior of the energy. However, this modification is still insufficient to get a fair agreement of *D*_e_ with the experimental value [7].

The molecular constant, *R*_e_, from Ref. [8] and our value (Table 1), lie on the opposite sides of the experimental value [7]. This can be related to the difference in the treatment of correlation in the two approaches. In our study we apply Sturm’s wave functions in the CI expansion. This is a very efficient treatment of correlation effects in atoms and molecules, because the purely discrete spectrum of Sturm’s eigenvalues contains the continuum spectrum as well and hence the CI expansion converges quickly. On the other hand, the *K*-functional technique of Ref. [8], makes use of an effective potential obtained by modifying the exchange operator in the Hamiltonian. This modification does not effect the closed shells of the interacting atoms therefore the polarization and correlation effects of the core are not taken into account. Finaly, we conclude that the use of the Sturm’s CI expansions for all orbitals of the OH molecule gives us an advantage in calculating the equilibrium internuclear distance *R*_e_.

The CI treatment of the AgH^+^ molecule excludes all closed orbitals of the Ag^+^ ion with the exception of the 4*p*^6^4*d*^10^ shells. Single- and double-excitations of the 4*p*^6^ and 4*d*^10^ shells are considered. Additional Sturm’s functions for 5*s*, 5*p*, 5*d*, 6*s*, and 6*p* orbitals were constructed for the CI expansion. The number of configurations is 150. Our calculated values for the molecular constants of AgH^+^ are given in Table 2. The other theoretical results [10] are obtained by using a DF pseudopotential and a density functional approach to describe the valence correlation and polarization energies of the molecule. Due to the fact that AgH^+^ is a “single” electron molecule, the correlation effects are not very important. However, the presence of the hydrogen atom polarizes the Ag^+^ ion orbitals and the CI treatment is still required. The comparison with other theoretical results [10] shows that we agree very well in the calculation of dissociation energies and mostly disagree in the computation of the rotational constant *ω*_e_.

## 3. Conclusions

In this paper we have shown that a quite accurate determination of molecular constants of molecules with open shells using our version of the valence-bond method is possible. We treated O and Ag as many-electron atoms, where core polarization must be properly accounted for. The disagreement with experimental values varies from 0.2 % to 4 % for the molecular parameters of OH. In addition the calculation of OH with a small number of configurations (38), which requires a few seconds of computer time for each internuclear separation, has less than a 1 % discrepancy for the *R*_e_ and *B*_e_ parameters. The difference between our values and other theoretical values for the AgH^+^ molecule does not exceed 7 %, except for *ω*_e_, which also serves as a further test of our method.

## Figures and Tables

**Table 1:** Constants of the X^2^∏ OH molecule (1 Å = 0.1 nm; the energy equivalent 1 cm^−1^ is 29.979 245 8 GHz; HF is the Hartree-Fock method)

	Basis set (number of conf.)	*R*_e_ (Å)	*ω*_e_ (cm^−1^)	*B* (cm^−1^)	*D*_e_ (eV)
This work	38	0.972	7297.25	18.782	−3.1701
	262	0.967	3638.39	18.834	−4.4302
HF+*K*-functional [8]		0.947			−4.4082
Experiment [7]		0.969	3737.76	18.910	−4.6259

**Table 2:** Constants of the X^2^Σ AgA^+^ molecule (DF is the Dirac-Fock method)

	Basis set (number of conf.)	*R*_e_ (Å)	*ω*_e_ (cm^−1^)	*B* (cm^−1^)	*D*_e_ (eV)
This work	150	2.19	366.14	3.5172	−0.145
DF [10]		2.37	515		−0.15
DF+core [10]		2.41	519		−0.14
